# Evaluation of Regional Lung Function in Pulmonary Fibrosis with Xenon-129 MRI

**DOI:** 10.3390/tomography7030039

**Published:** 2021-09-15

**Authors:** Jaime Mata, Steven Guan, Kun Qing, Nicholas Tustison, Yun Shim, John P. Mugler, Talissa Altes, Jhosep Huaromo, Borna Mehrad

**Affiliations:** 1 Department of Radiology and Medical Imaging, University of Virginia, Charlottesville, VA 22901, USA; sg8uq@virginia.edu (S.G.); kq4d@virginia.edu (K.Q.); ntustison@virginia.edu (N.T.); yss6n@virginia.edu (Y.S.); John.Mugler@virginia.edu (J.P.M.III); jeh3scy@virginia.edu (J.H.); 2 Department of Medicine, University of Virginia, Charlottesville, VA 22901, USA; 3 Department of Radiology, University of Missouri, Columbia, MO 65211, USA; altest@health.missouri.edu; 4 Department of Medicine, University of Florida, Gainesville, FL 32611, USA; sara.mayo@medicine.ufl.edu

**Keywords:** interstitial lung disease, idiopathic pulmonary fibrosis, hyperpolarized Xenon-129, MRI

## Abstract

Idiopathic pulmonary fibrosis, a pattern of interstitial lung disease, is often clinically unpredictable in its progression. This paper presents hyperpolarized Xenon-129 chemical shift imaging as a noninvasive, nonradioactive method of probing lung physiology as well as anatomy to monitor subtle changes in subjects with IPF. Twenty subjects, nine healthy and eleven IPF, underwent HP Xe-129 ventilation MRI and 3D-SBCSI. Spirometry was performed on all subjects before imaging, and DLCO and hematocrit were measured in IPF subjects after imaging. Images were post-processed in MATLAB and segmented using ANTs. IPF subjects exhibited, on average, higher Tissue/Gas ratios and lower RBC/Gas ratios compared with healthy subjects, and quantitative maps were more heterogeneous in IPF subjects. The higher ratios are likely due to fibrosis and thickening of the pulmonary interstitium. T2* relaxation was longer in IPF subjects and corresponded with hematocrit scores, although the mechanism is not well understood. A lower chemical shift in the red blood cell spectroscopic peak correlated well with a higher Tissue/RBC ratio and may be explained by reduced blood oxygenation. Tissue/RBC also correlated well, spatially, with areas of fibrosis in HRCT images. These results may help us understand the underlying mechanism behind gas exchange impairment and disease progression.

## 1. Introduction

Interstitial lung diseases (ILD) are a group of complex diseases characterized by various patterns of inflammation and fibrosis affecting the alveolar, interstitial, and vascular compartments of the lung [1]. ILD can be classified by its numerous causes, such as hypersensitivity pneumonitis, connective tissue disease, pneumoconiosis, drug toxicities or radiation exposure. ILD with no identifiable cause is commonly due to idiopathic pulmonary fibrosis (IPF), one of the most aggressive forms of ILD. Patients with IPF often have other comorbid conditions, including emphysema, lung cancer, and pulmonary hypertension [2]. A recent systematic review investigating the incidence of IPF estimated it in Europe and North America to be 3 to 9 cases per 100,000 per year. In East Asia and South America, the incidence was lower [3]. The mortality of IPF is high. There is a median survival of 2–3 years after diagnosis, and evidence shows survival has not been improving over the last several years. In fact, mortality rates seem to be rising, though this could be due to increased awareness and knowledge of IPF [4].

Our current understanding of the pathogenesis of IPF posits that there is an abnormal repair process of injured alveoli in the lungs. Oxidative injury, mitochondrial dysfunction, and shortened telomeres can result in decreased proliferation of alveolar epithelial cells and release of profibrotic mediators. These mediators, such as platelet-derived growth factor (PDGF) and tumor necrosis factor-beta (TGF-β), can stimulate matrix deposition by myofibroblasts. Accordingly, IPF shows a characteristic pattern termed usual interstitial pneumonia (UIP) on lung biopsy. This pattern describes heterogenous paraseptal fibrosis with remodeling of the lung architecture. On high-resolution computed tomography (HRCT), the UIP pattern consists of reticulation and honeycombing in the peripheral regions of the lungs and in the lower lobes [5].

No cure for IPF exists. The current management of IPF involves attempting to slow disease progression. Two medications that have shown some evidence in accomplishing this include the antifibrotic agents Nintedanib and Pirfenidone, both of which have adverse effects that must be considered when choosing the best option for a patient. On the other hand, anti-acid therapy has fewer adverse effects, and one study showed it may increase survival in IPF patients. However, it is important to note that anti-acid therapy has not been studied in randomized trials [6]. 

A critical barrier to progress in the field is the unpredictability of the clinical course of interstitial lung diseases: for example, the rate of decline in lung function is highly heterogeneous in IPF [7]. Furthermore, the progression IPF is often episodic, characterized by prolonged periods of relative stability, and abrupt and unpredictable acute exacerbations that are associated with markedly worsened prognosis [8]. Developing tools for prior identification of patients at risk for disease progression would constitute a major advancement in the field by allowing targeting of such at-risk populations for currently approved antifibrotic therapies and for trials of novel agents. Achieving an earlier diagnosis using more sensitive technologies has the additional potential to allow for patients to receive earlier treatment [9]. Finally, the stratification of ILD patients is a necessary step toward the development of more effective and personalized treatments [10]. Currently, HRCT, lung biopsy, and spirometry tests are the main techniques used for the diagnosis of IPF, for classifying disease severity, and for assessing the efficacy of antifibrotic therapies [2]. However, these techniques entail radiation exposure, are invasive, or can only examine global lung function. 

Hyperpolarized Xenon-129 magnetic resonance imaging (HP Xe-129 MRI) is a promising candidate for fulfilling these unmet needs. It has enabled many approaches, such as ventilation and diffusing imaging, for the evaluation of lung structure and function [11,12,13]. More specifically, xenon has several unique properties that are well suited for probing regional gas exchange impairment in IPF. Xenon is sensitive to its chemical environment, produces an enormous range of chemical shifts, and has a high solubility in biological tissues [14,15]. Upon inhalation, approximately 2% of the gaseous xenon dissolves into the surrounding pulmonary tissue, plasma, and red blood cells (RBC). The gaseous and dissolved xenon produces a set of detectable MR spectral peaks at distinct frequencies that can be measured and quantified [16,17]. Three peaks are typically observed in human Xe-129 lung spectra, corresponding to gaseous xenon in the airspaces (0 ppm), xenon dissolved in the interstitium (197 ppm), and xenon associated with hemoglobin in the blood (217 ppm).

To exploit these properties of xenon, we developed an MR spectroscopic imaging technique termed 3D Single-Breath Chemical Shift Imaging (3D-SBCSI). This technique is capable of non-invasively assessing regional lung ventilation and gas exchange within a single breath hold of less than 10 s [18,19,20,21,22,23,24]. Previous studies in animal lung fibrosis models have demonstrated that tissue thickening can be detected using 3D-SBCSI [21,22,23,24]. 

Because lung anatomy, ventilation, and gas exchange can be examined by 3D-SBCSI, radiation exposure that comes with HRCT can be avoided, and the assessment of regional lung function rather than global lung function can be achieved in a non-invasive manner. In this study, we aimed to determine how effective 3D-SBCSI is in detecting regional lung function and structural changes in patients with IPF by correlating those findings with spirometry tests and HRCT scores. 

## 2. Materials and Methods

This study was performed under a protocol approved by the Institutional Review Board and an investigational new drug application for Xe-129 MRI. Written informed consent was obtained from all participants. A total of 20 subjects, including 9 healthy (29 yo ± 9.2; FEV1_pred_ = 99 ± 9.9%; FVC_pred_ = 104 ± 10.6%) and 11 who met diagnostic criteria for IPF (65 yo ± 12.5; FEV1_pred_ = 63 ± 13.9%; FVC_pred_ = 63 ± 15.2%) underwent HP Xe-129 ventilation MRI and 3D-SBCSI (Table 1). Spirometry was performed on all subjects before imaging. Diffusion capacity for carbon monoxide (DLCO) and hematocrit were measured only in IPF subjects after imaging. 

All imaging protocols were performed on a 1.5 T clinical MRI system (Avanto, Siemens Medical Solutions, USA) using a transmit/receive RF coil (Clinical MR Solutions, WI) tuned to the Xe-129 frequency. Subjects were placed supine on the MR table with the coil strapped to their chest. 3D proton localizer MR images were used to position the subject’s lungs at the isocenter. A test dose of xenon was well tolerated by all subjects, and there were no signs of any adverse reactions. Isotopically enriched (~83%) Xe-129 was polarized to ~35% using a commercial Xe-129 polarizer. For each Xe-129 MRI acquisition, the subjects inhaled a gaseous mixture of HP Xe-129 (maximum volume of 1.0 L) and nitrogen for a total volume equal to one-third of their measured FVC. Immediately after inhalation, the subjects were asked to hold their breath for the entire duration of the acquisition (<10 s). The 3D-SBCSI sequence parameters used were: TR/TE 13 ms/1.0 ms; FA 25° centered at 200 ppm of the gas frequency; vector size 512; weighted phase-encoding; BW 50 kHz; minimum voxel size 6.5 × 6.5 mm^2^; and 6–8 slices with 20–25 mm slice thickness. 

H1-MRI images, covering the whole lungs with 17 slices and using a pulse sequence with a spiral K-space trajectory, were obtained during the same breath hold as HP Xe-129 Gradient Echo (GRE) images, from which we created a mask for quantifying relative ventilation in the GRE images. The intensity of the GRE images corresponds to the amount of HP Xe-129 detected in a particular voxel and can be used to measure relative ventilation in each voxel, segmenting the GRE images into four classifications: hyperventilation, normal ventilation, hypoventilation, and no ventilation. This analysis was completed using the Advanced Normalization Tools (ANTs) package, as described elsewhere [25]. 

Following the segmentation of the GRE images, the volumes of three classifications were determined; the two lower ventilation classifications (no ventilation and hypoventilation) correspond to ventilation defects, while the higher ventilation classification indicates normal ventilation. These classification volumes were normalized using the sum of each of the classification volumes, the total measured volume of the lung, for each particular subject, calculating the percentage of ventilation defects and normal ventilation in the total volume of each subject’s lungs.

Post-processing of 3D-SBCSI data was completed with custom software written in MATLAB (MathWorks, Natick, MA, USA), which allowed the production of the desired spectrum for quantification. Traditionally, a peak in a spectrum is quantified by calculating the peak’s area under the curve by directly integrating the spectrum within specified boundaries. However, a spectrum with overlapping peaks such as the Xe-129 spectrum cannot be directly integrated and requires additional processing for accurate quantification [26,27]. For this reason, the HP Xe-129 complex spectrum was fitted to a sum of complex Lorentzian functions. Each peak was individually modeled by a Lorentzian function that accounted for the peak’s amplitude, width, chemical shift, and phase [28,29]. Subsequently, the area under the curve was calculated for each peak using its respective fitted parameters.

The quantification process was repeated for every acquired voxel to generate maps describing the relative amounts of xenon in the Gas, Tissue, and RBC compartments. To allow for more meaningful and normalized comparisons, Tissue/Gas, RBC/Gas, and Tissue/RBC ratio maps were calculated from the component maps. In addition to the ratio maps, the fitted parameters were also used to generate maps describing the chemical shift and T_2_* of the Tissue and RBC peaks. 

HRCT evaluations were acquired only for IPF subjects because unnecessary exposure of healthy subjects to ionizing radiation was not ethically justifiable. HRCT images were evaluated for reticulation, traction bronchiectasis, ground-glass opacities, honeycombing, and emphysema at three different regions of each lung (above the aortic arch, between the aortic arch and pulmonary veins, and below the pulmonary veins), using a validated scoring system [30,31]. A whole lung score for each radiographic feature was calculated by averaging of the six regional scores. Each HRCT was independently scored by two experienced radiologists, with a good agreement between the two (Krippendorf’s alpha = 0.80).

Statistical analysis was performed for each 3D-SBCSI parameter on a whole lung and on an individual slice level. The Mann–Whitney U-test was used to determine significance between healthy and IPF subjects. A two-tailed Pearson correlation was used to determine if there was a statistically significant correlation between 3D-SBCSI, pulmonary function tests (PFT), and HRCT. 

## 3. Results

### 3.1. Comparison of Healthy and IPF Ventilation Images

**Figure 1:**
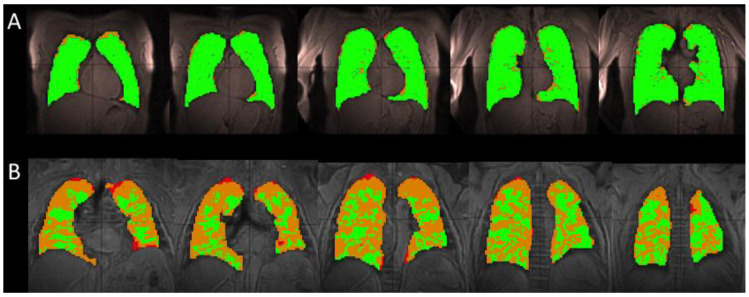
A comparison of the segmented ventilation images for a single healthy (H2) (**A**) and IPF (I2) subject (**B**). The healthy subject has mainly areas of normal (green) ventilation, with hypoventilation (orange) and no ventilation (red) areas located on the periphery of the lung, occupying 4.8% of the total lung volume for this particular subject. In the IPF subject, these large ventilation defects located on the periphery of the lung encroach toward the center of the lung, occupying 35.1% of the total lung volume. The IPF subject retained a portion of the inhaled xenon in the trachea and bronchi, resulting in the high intensity of gas in those areas (not shown). The segmentation algorithm excluded these regions to avoid overestimating the volume of the lungs and areas of ventilation.

Subjects with IPF had significantly increased volumes of ventilation defects (VD), defined as volumes with no ventilation at all, plus volumes with hypoventilation or lower SNR, as compared with healthy subjects. Healthy subjects had 9.6 ± 7.37% VD, while IPF subjects had 34.1 ± 6.44% VD (*p* < 0.01; Figure 1). Additionally, healthy subjects had an average FVC of 4.6 ± 0.91 L and total lung volume calculated from the ventilation images equal to 4.1 ± 0.56 L. IPF subjects had lower spirometry FVC values, 2.4 ± 0.87 L, compared with the total volume calculated from the ventilation images, 2.6 ± 0.70 L. In both groups, spirometry measurements had higher standard deviations.

The % VD correlated strongly with age (R = 0.74), Tissue/RBC ratio (R = 0.74), RBC chemical shift (R = −0.83), and RBC-Tissue chemical shift (R = −0.80).

### 3.2. Comparing Ratio Maps between Healthy and IPF

As seen in the segmented Xe-129 ventilation MR images in Figure 1A, the lungs of healthy subjects were well ventilated and had few ventilation defects. IPF subjects had multiple ventilation defects predominantly located at the lung periphery, as seen in Figure 1B. 

**Figure 2:**
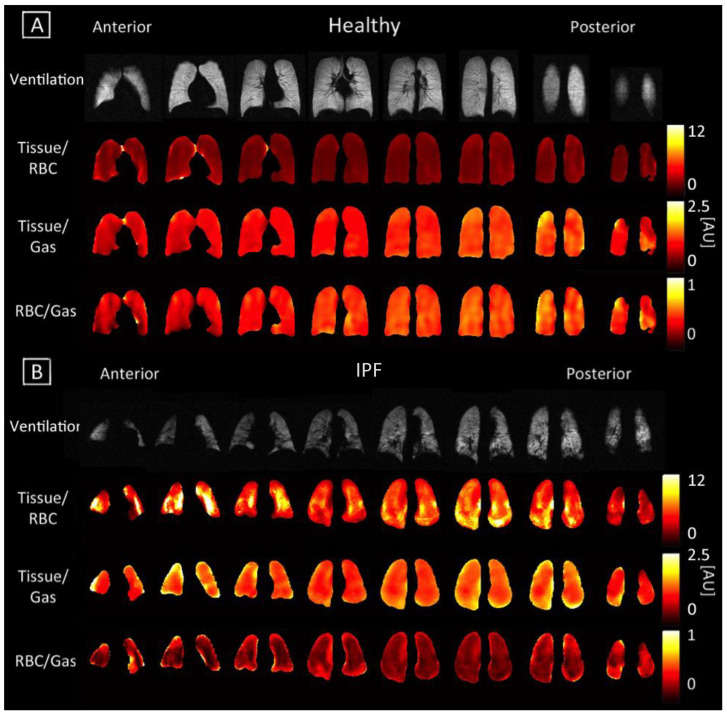
Coronal maps of ventilation and 3D-SBCSI Tissue/RBC, Tissue/Gas, and RBC/Gas in (**A**) healthy subject H4 and (**B**) IPF subject I8.

**Figure 3:**
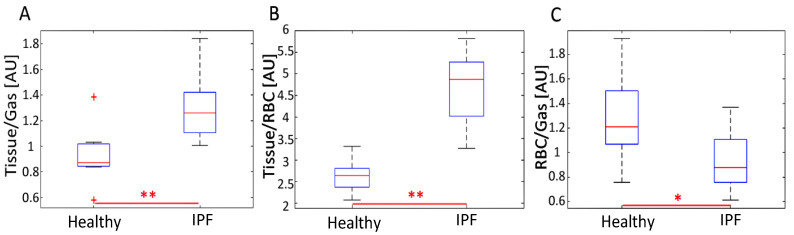
Distribution of average (**A**) Tissue/Gas, (**B**) Tissue/RBC, and (**C**) RBC/Gas ratios for healthy and IPF subjects. IPF subjects typically had a significantly increased average Tissue/Gas and Tissue/RBC ratio (*p* < 0.01) and a decreased average RBC/Gas ratio (*p* < 0.05). The distribution of average Tissue/RBC ratios was larger in IPF than healthy subjects. The uppermost outlier in (**A**) represents subject H8. * represents *p* < 0.05 and ** represents *p* < 0.01.

Gravity-dependent gradients from the anterior to posterior direction were observed in the Tissue/Gas and RBC/Gas maps of all subjects, as previously reported elsewhere [32]. These gradients were more pronounced and had smoother transitions between coronal slices in healthy subjects (Figure 2), while they appeared to vary in IPF subjects. The average Tissue/Gas ratio was significantly higher in IPF subjects compared with healthy subjects (1.31 ± 0.26 versus 0.93 ± 0.22, *p* < 0.01; Table 2, Figure 2 and Figure 3A); conversely, the average RBC/Gas ratio was significantly lower in IPF subjects compared with healthy subjects (0.28 ± 0.06 versus 0.37 ± 0.09, *p* < 0.05; Table 2, Figure 2 and Figure 3C).

Anterior to posterior gravity-dependent gradients were prominent in Tissue/RBC maps since an increase in the Tissue/Gas ratio coincided with a similar increase in the RBC/Gas ratio. Gradients were still more pronounced in healthy subjects than in IPF subjects, as seen in Figure 2. In healthy subjects, the Tissue/RBC maps had a relatively homogenous distribution, in which the center of the lungs tended to have lower ratio values due to slightly higher values in the RBC/Gas. In IPF subjects, the Tissue/RBC maps had a highly heterogeneous distribution with regions of exceptionally high ratios at the periphery and base of the lungs (Figure 2). The average Tissue/RBC was significantly higher in IPF subjects than in healthy subjects, (4.71 ± 0.81 versus 2.63 ± 0.37, *p* < 0.01; Table 2, Figure 3B).

### 3.3. Comparing T2* and Chemical Shift Maps

**Figure 4:**
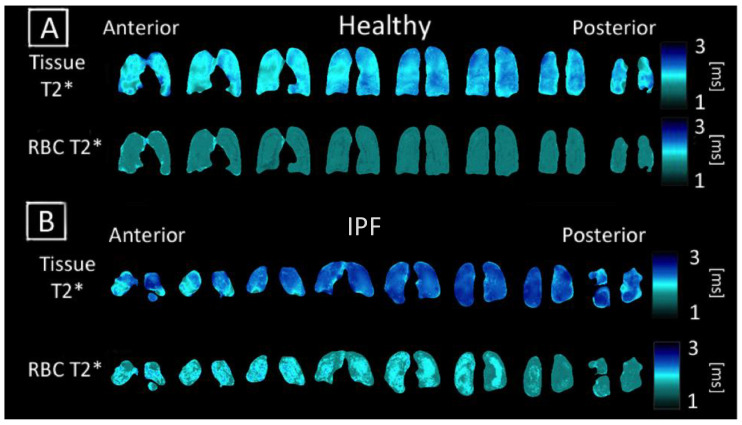
Coronal maps of 3D-SBCSI Tissue T2*, RBC T2* in (**A**) healthy subject H4 and (**B**) IPF subject I1.

**Figure 5:**
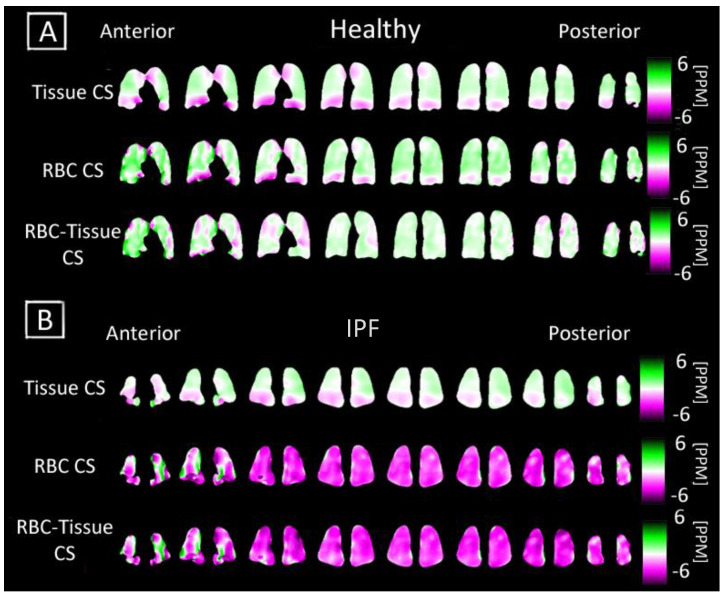
Coronal maps of 3D-SBCSI Tissue Chemical Shift (CS), RBC Chemical Shift in a (**A**) healthy subject H4 and (**B**) IPF subject I9. The respective chemical shift maps were normalized to show how each voxel deviated from their expected tissue chemical shift (197 ppm) and RBC chemical shift (216 ppm).

Unlike direct dissolved-phase imaging techniques, 3D-SBCSI can regionally probe and generate maps describing these T2* (measurement of Xe-129 relaxation time) and chemical shift parameters shown in Figure 4 and Figure 5 [33]. The Tissue and RBC chemical shift maps typically had a lower chemical shift of 1–2 ppm at the base of the lungs in all subjects. 

**Figure 6:**
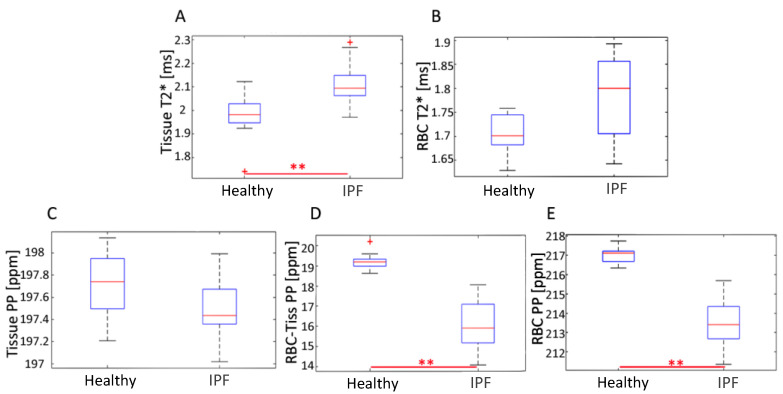
Distribution of average (**A**) Tissue T_2_*, (**B**) RBC T_2_*, (**C**) Tissue CS, (**D**) RBC-Tissue CS and (**E**) RBC CS for healthy and IPF subjects. ** represents *p* < 0.01.

Regions with higher values in the RBC T2* map corresponded with regions of higher Tissue/RBC ratios. However, regional variations in the RBC T2* maps did not account for all heterogeneities observed in the Tissue/RBC maps. IPF subjects had a significantly larger average Tissue T2*, 2.12 ± 0.09 ms, than healthy subjects, 1.97 ± 0.11 ms, (*p* < 0.01) (Table 2, Figure 6). Similarly, the average RBC T2* was larger in IPF subjects, 1.79 ± 0.09 ms, than in healthy subjects, 1.71 ± 0.04 ms (*p* = 0.06) (Table 2, Figure 6). The tissue chemical shift was not significantly different between healthy and IPF subjects, but the RBC chemical shifts were significantly different (*p* < 0.01) (Figure 6C–E). There was also a very strong correlation between the Tissue/RBC ratio and RBC chemical shift for all subjects (R = 0.97).

### 3.4. Subject Characterization with PFTs and HRCT

As expected, the FVC was significantly lower in IPF subjects, 66.45 ± 15.02%, than in healthy subjects, 103.4 ± 10.57%, (*p* < 0.01). The FEV1 predicted was also significantly lower in IPF subjects, 67.5 ± 14.68%, than in healthy subjects, 99.3 ± 10.00%, (*p* < 0.01) (Table 2). 

Scores for reticulation, traction bronchiectasis, ground glass opacity, honeycombing, and emphysema in the HRCT images of IPF subjects were calculated and summarized for the whole lung (Table 2) and regionally (Table 3). There was a good agreement between the two readers scoring the HRCT images (Krippendorf’s alpha = 0.80). As expected, the lower lobes had higher scores for all radiographic features except for emphysema [34].

### 3.5. Correlation between 3D-SBCSI with PFTs and HRCT

**Figure 7:**
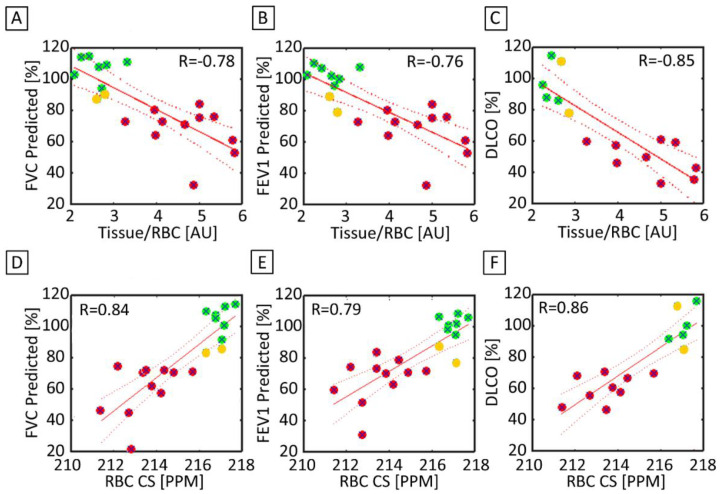
(**A**) FVC predicted and the Tissue/RBC ratio were significantly and strongly correlated between healthy (green), healthy but low PFT (yellow), and IPF (red) subjects (R = −0.78). (**B**) There was also a significant strong correlation between FEV1 predicted and the Tissue/RBC ratio (R = −0.76). (**C**) DLCO and the Tissue/RBC ratio (R = −0.85) were also significantly strongly correlated. (**D**) FVC predicted and the RBC chemical shift were significantly correlated (R = 0.84). (**E**) There was another significant and strong correlation between FEV1 predicted and the RBC chemical shift (R = 0.79). (**F**) DLCO and the RBC Chemical Shift (R = 0.86) were also significantly correlated. The solid line represents a linear regression of the data, while the dashed lines shows the 95% confidence interval of the linear regression.

The Tissue/RBC ratio and FVC were significantly correlated (R = −0.78, *p* < 0.01) for both the healthy and IPF subjects, as seen in Figure 7A. Tissue/RBC ratio and FVC did not correlate within the healthy population (R = 0.10) but were inversely correlated within the IPF population (R = −0.69). The Tissue/RBC ratio and FEV1 were also significantly correlated (R = −0.76, *p* < 0.01) for our healthy and IPF population (Figure 7B). There was a very good correlation between Tissue/RBC ratio and DLCO (R = −0.85, *p* < 0.01) (Figure 7C). 

**Figure 8:**
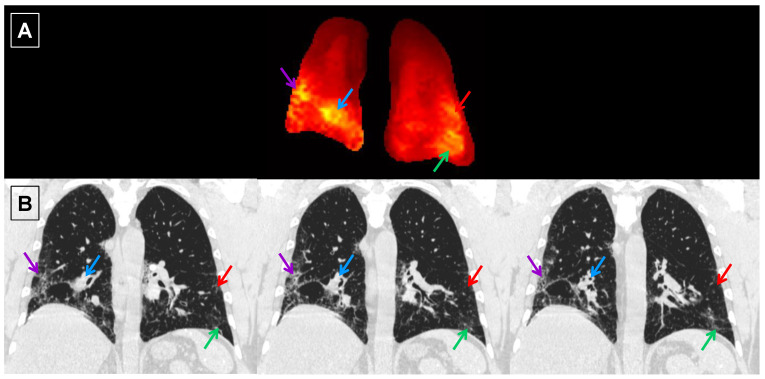
Central coronal slice of the Tissue/RBC maps (**A**) and spatially matched coronal HRCT images (**B**) from IPF subject I6. Due to the difference in the slice thickness between the Tissue/RBC maps (15 mm) and HRCT images (0.7 mm), information from multiple nearby HRCT images could appear in a single slice of the Tissue/RBC maps. Each arrow points to a region of high Tissue/RBC, and the same color arrow points to the corresponding region of fibrosis in the HRCT images.

In order to correlate the HRCT regional findings with physiological maps from the 3D SBCSI, the CSI lung maps and data (Table 4) were divided into regions corresponding to the areas scored in HRCT (upper, middle, and lower thirds of lungs). Among the HRCT findings, RBC CS had the strongest correlation (R = −0.99) with all HRCT scores except emphysema, and there were very strong correlations among HRCT scores and Tissue CS, RBC-Tissue CS, Tissue T2*, and RBC T2* as well (Table 5). Furthermore, regions of high Tissue/RBC values at the base and periphery of the lungs were found to correspond well to regions of fibrosis in the HRCT images (Figure 8).

## 4. Discussion

### 4.1. Ventilation Images

The volume of ventilation defects was significantly higher in our IPF subjects, especially in the periphery of the lungs (Figure 1). The subjects are unable to fully ventilate their lungs, which is evidenced by strong correlations (R > 0.62) between percent volume of ventilation defects and all spirometric measurements (FEV1, FVC, FVC predicted, and FEV1% predicted). The ventilation images reveal specific regions of the lung that experience poor ventilation, providing a base framework for the CSI maps when considering underlying causes of ventilation defects. The volume of ventilation defects also correlated strongly with Tissue/RBC ratio, RBC CS, and RBC-Tissue CS. 

### 4.2. Ratio Maps

The average Tissue/RBC ratio was significantly higher in IPF subjects than in healthy subjects, revealing that IPF subjects suffered from impaired gas exchange (Table 2, Figure 3B). Interestingly, the severity of gas exchange impairment was not homogenous throughout the lungs as demonstrated by the highly heterogeneous distribution of Tissue/RBC ratios (Figure 2B). Regions at the periphery and base of the lungs typically had higher ratio values, suggesting these areas suffered from more severe gas exchange impairment. These regional heterogeneities could serve as markers for tracking disease progression and monitoring severity in IPF. 

IPF subjects exhibited higher Tissue/Gas ratios and lower RBC/Gas ratios than healthy subjects, likely due to fibrosis and thickening of the pulmonary interstitium (Table 2, Figure 3A,C). A thicker interstitium allowed more xenon to dissolve into the tissue, leading to a higher Tissue/Gas ratio. The thickened tissue prevented xenon from reaching the RBC compartment, resulting in a lower RBC/Gas ratio. 

These results are consistent with the results and conclusions by Wang et al. [35], indicating that our findings are rooted in physiology and anatomy rather than in a particular subject pool, imaging technique, or analysis method. Discrepancies in our results are likely due to population differences, not method sensitivity.

The percent of ventilation defects in healthy subjects was slightly higher than expected. This is a result of coil drop-off at the upper lobes of some healthy subjects, resulting in peripheral lung voxels to be classified as regions of hypoventilation rather than normal regions.

### 4.3. Tissue and RBC T2* Maps

Additional insights into pulmonary function such as regional tissue thickness, tissue heterogeneity, and blood oxygenation could be gleaned from analyzing the chemical shift and T2* of the Tissue and RBC peaks. Currently, the physiological meaning of changes in the chemical shift and T2* of the Tissue and RBC peaks have not been well-studied and are not fully understood. Norquay et al. [26] found that the blood oxygenation level is strongly correlated with the RBC chemical shift. The RBC peak shifted further away from the Gas peak as the blood oxygenation level increased [26,27]. Furthermore, Wolber et al. [36] have reported that the T2* of HP Xe-129 in plasma decreased linearly with hematocrit, which was hypothesized to be caused by increasingly frequent xenon exchange between the plasma and RBCs. There were no previous reports of physiological factors influencing the Tissue T2*, chemical shift, and the RBC T2*. Nevertheless, these reported studies provided a basis for interpreting the physiological meanings of the 3D-SBCSI chemical shift and T2* maps.

Regional heterogeneities observed in the RBC T2* maps tended to correspond well with those observed in the Tissue/RBC maps seen in Figure 2 and Figure 4, suggesting that a change in the RBC T2* was linked with changes in the Tissue/RBC ratio. Nevertheless, the RBC T2* and Tissue/RBC were not significantly correlated (*p* = 0.13), and variations in the RBC T2* maps did not account for all of the heterogeneities observed in the Tissue/RBC maps. Thus, some combination of changes in the Tissue T2* and amplitudes of the Tissue and RBC peaks were also responsible for the observed heterogeneities and increased Tissue/RBC ratios in IPF subjects. While a high Tissue/RBC ratio indicated impaired gas exchange, there could be multiple underlying mechanisms that led to the impairment. By identifying the combination of factors leading to increased Tissue/RBC ratios, it could be possible to differentiate between mechanisms causing impaired gas exchange.

The average Tissue T2* was significantly larger in IPF subjects, while the average RBC T2* was larger but not significant (Table 2, Figure 4A,B and Figure 6A,B). The significantly increased Tissue T2* in IPF could be caused by the decrease in susceptibility effects as a result of the decreased blood oxygenation and thicker fibrotic tissues shielding the xenon from magnetic susceptibilities from air-tissue interfaces.

### 4.4. Correlations with PFTs and HRCT

Tissue/RBC ratio and FVC had a strong correlation (R = −0.78) and were able to detect changes in pulmonary function and clearly discriminate between healthy and IPF subjects, as shown in Figure 7A. As expected, there was a poor correlation among healthy subjects since each technique was probing fundamentally different phenomena: Tissue/RBC is a measurement of gas exchange while FVC is a measurement of ventilation. There was a good correlation within IPF subjects as pathological alterations in lung structure affecting ventilation would also affect gas exchange. While differences between healthy and IPF subjects could be seen in both parameters, the Tissue/RBC ratio was a more sensitive parameter. For example, within the IPF subjects, there were six subjects with similar FVCs ranging from 76% to 79%, while their average Tissue/RBC ratio ranged from 3.27 to 5.36 (Figure 7A). This increased sensitivity over PFTs and ability to detect physiologic discrepancies that go unnoticed in traditional PFTs would allow for an improved measurement of pulmonary function for diagnosis and disease management.

Two of the healthy subjects had slightly abnormal PFT results (yellow dots on Figure 7). The 3D-SBCSI data obtained from these two subjects were also out of the range when compared with the other healthy subjects in their group. This tells us that these two subjects are not as healthy and that our technique may be useful in detecting early stages of lung disease.

There was a very strong correlation between Tissue/RBC ratio and DLCO (Figure 7C) despite measuring different gases in different lung compartments, and this is consistent with the strong correlation between these two parameters found in the studies by Wang et al. [35] and Kaushik et al. [28]. The presence of fibrotic and thickened tissues in IPF could have affected measurements of gas exchange unequally in each technique. 

Ground-glass opacity scores (R = −0.40), including other HRCT scores, were not strongly correlated with the Tissue/RBC ratio, as shown in Table 5, which is consistent with the correlation between fibrosis scores and Tissue/RBC ratio found by Wang et al. [36]. However, regions of high Tissue/RBC ratios were well matched with regions of fibrosis in HRCT images (Figure 8). This agreement supports the observation that regional heterogeneities indicating gas-exchange impairment in Tissue/RBC maps were linked to fibrosis observed in HRCT images.

### 4.5. Study Limitations

The total number of subjects that underwent HP Xe-129 MR imaging in this study was relatively small and may not be representative of the larger population of subjects with IPF. In addition, the IPF and healthy subjects were not age-matched, and some of the differences observed may be confounded by age.

## 5. Conclusions

HP Xe-129 3D-SBCSI is a promising clinical tool that has been shown to be capable of non-invasively assessing regional lung ventilation and gas exchange. By acquiring the full HP Xe-129 spectrum for each voxel, 3D-SBCSI provides unique spectral information, such as the chemical shift and T2* of the Tissue and RBC peaks, which can be potentially exploited to gain further insights into regional blood oxygenation and mechanisms of gas exchange impairment. Due to this information acquired from 3D-SBCSI, its application to other pulmonary diseases, including cystic fibrosis in pediatric patients and lung cancer, has been studied and is still ongoing [18,19,20,37]. 

We found that IPF subjects exhibited higher Tissue/Gas ratios and lower RBC/Gas ratios compared with healthy subjects, most likely due to the fibrosis and thickening of the pulmonary interstitium with a peripheral and basilar predominance and good correlation with PFTs and HRCT features. Future work is required to gain a better understanding of the physiological implications of these parameters. Longitudinal studies with a larger population of subjects with different types of IPF are also needed to validate the findings of this study. Nevertheless, having this novel regional physiological information from 3D-SBCSI in addition to HRCT could allow for a potentially earlier diagnosis of IPF and the stratification of IPF patient types leading to improved treatments and clinical outcomes.

## Figures and Tables

**Table 1:** Subject data for healthy (*n* = 9) and IPF (*n* = 11) subjects.

Subject No.	Diagnosis	Sex	FVC Pred. [%]	FEV1 Pred. [%]	Age (Years)
H1	Healthy	M	103	103	26
H2	Healthy	F	115	107	29
H3	Healthy	F	111	108	52
H4	Healthy	F	108	102	24
H5	Healthy	M	90	79	31
H6	Healthy	M	114	110	29
H7	Healthy	M	109	100	24
H8	Healthy	F	87	89	21
H9	Healthy	M	94	96	24
I1	IPF	M	76	84	74
I2	IPF	M	77	80	70
I3	IPF	M	76	73	67
I4	IPF	F	31	32	38
I5	IPF	M	77	75	80
I6	IPF	M	79	76	68
I7	IPF	M	76	73	56
I8	IPF	M	64	64	68
I9	IPF	F	53	53	64
I10	IPF	M	68	71	64
I11	IPF	F	54	61	79

**Table 2:** Summary of whole lung averages for healthy and IPF subjects.

		Healthy	IPF	*p*-Value
**3D-SBCSI**	Tissue/RBC	2.63 ± 0.37	4.71 ± 0.81	<0.01
	Tissue/Gas	0.93 ± 0.22	1.31 ± 0.26	<0.01
	RBC/Gas	0.37 ± 0.09	0.28 ± 0.06	<0.05
	Tissue T_2_* [ms]	1.97 ± 0.11	2.12 ± 0.09	<0.01
	RBC T_2_* [ms]	1.71 ± 0.04	1.79 ± 0.09	0.06
	Tissue Chemical Shift [PPM]	197.7 ± 0.29	197.5 ± 0.29	0.07
	RBC Chemical Shift [PPM]	217.0 ± 0.44	213.5 ± 1.25	<0.01
	RBC-Tissue Chemical Shift [PPM]	19.2 ± 0.45	16.1 ± 1.21	<0.01
**PFT**	FEV1 [%]	99.3 ± 10.00	67.5 ± 14.68	<0.01
	FVC [%]	103.4 ± 10.57	66.5 ± 15.02	<0.01
	DLCO [%]	96 ± 14.7	48 ± 12.1	<0.01
	Hematocrit [%]	43 ±1.5	41 ± 2.3	0.07
**HRCT Score**	Reticulation	-	29 ± 13.7	-
	Traction Bronchiectasis	-	15 ± 12.8	-
	Ground-Glass opacities	-	21 ± 10.6	-
	Honeycombing	-	12 ± 10.8	-
	Emphysema	-	0.8 ± 1.5	-
**Ventilation**	Total Volume [L]	4.1 ± 0.56	2.6 ± 0.70	<0.05
	Relative Defect Volume [%]	9.6 ± 7.37	34.1 ± 6.44	<0.05

**Table 3:** HRCT radiographic findings (Score) by lung region.

	Reticulation	Traction Bronchiectasis	Ground Glass Opacity	Honeycombing	Emphysema
**Right Upper**	20.5 ± 1.41	5.8 ± 2.47	12.8 ± 4.59	3.0 ± 0.71	3.3 ± 0.35
**Left Upper**	15.8 ±1.77	2.8 ± 1.77	8.8 ± 4.59	1.5 ± 2.12	3.3 ± 0.35
**Right Mid**	29.5 ± 1.41	12.8 ± 3.89	24.0 ± 0.00	12.3 ± 1.06	3.0 ± 0.71
**Left Mid**	27.8 ± 3.89	10.8 ± 2.47	19.5 ± 7.07	9.8 ± 0.35	3.0 ± 0.71
**Right Lower**	43.3 ± 7.42	28.3 ± 3.18	35.0 ± 7.01	26.3 ± 0.35	0.5 ± 0.81
**Left Lower**	41.3 ± 5.30	27.0 ± 0.00	32.3 ± 8.13	25.0 ± 0.00	0.5 ± 0.71

**Table 4:** 3D-SBCSI data by lung region for IPF subjects.

	Tissue/RBC [AU]	Tissue/Gas [AU]	RBC/Gas [AU]	Tissue T2* [ms]	Tissue-RBC T2* [ms]	Tissue CS [PPM]	RBC T2* [ms]	RBC-Tissue CS [PPM]	RBC CS [PPM]
**Right Upper**	4.88	1.37	0.28	2.18	0.36	197.72	1.81	16.19	213.84
**Left Upper**	4.92	1.50	0.32	2.17	0.36	197.82	1.82	16.18	213.92
**Right Mid**	4.71	1.24	0.27	2.10	0.32	197.40	1.78	16.18	213.49
**Left Mid**	4.65	1.40	0.31	2.11	0.30	197.53	1.81	16.15	213.64
**Right Lower**	4.86	1.22	0.26	2.10	0.38	197.28	1.75	16.00	212.93
**Left Lower**	4.71	1.41	0.30	2.08	0.35	197.35	1.78	15.93	213.14

**Table 5:** Correlation Coefficients (R) between HRCT scores and 3D SBCSI data by lung region (Table 3 and Table 4).

	Reticulation	Traction Bronchiectasis	Ground Glass Opacity	Honeycombing	Emphysema
**Tissue/RBC [AU]**	−0.39	−0.29	−0.40	-0.33	0.12
**Tissue/Gas [AU]**	−0.57	−0.51	−0.63	−0.52	0.34
**RBC/Gas [AU]**	−0.51	−0.47	−0.56	−0.47	0.35
Tissue T2* [ms]	−0.85	−0.80	−0.87	−0.84	0.67
Tissue-RBC T2* [ms]	0.23	0.34	0.20	0.29	−0.50
RBC T2* [ms]	−0.88	−0.87	−0.92	−0.87	0.77
**Tissue CS [PPM]**	−0.95	−0.91	−0.97	−0.93	0.78
**RBC-Tissue CS [PPM]**	−0.87	−0.91	−0.83	−0.90	0.97
**RBC CS [PPM]**	−0.99	−0.99	−0.99	−0.99	0.94

## Data Availability

Data available upon request.
